# The Impact of Oyster Mushrooms (*Pleurotus ostreatus*) on the Baking Quality of Rye Flour and Nutrition Composition and Antioxidant Potential of Rye Bread

**DOI:** 10.3390/foods14020199

**Published:** 2025-01-10

**Authors:** Sylwia Stępniewska, Agnieszka Salamon, Grażyna Cacak-Pietrzak, Małgorzata Piecyk, Hanna Kowalska

**Affiliations:** 1 Department of Food Technology and Assessment, Division of Fruit, Vegetable and Cereal Technology, Institute of Food Sciences, Warsaw University of Life Sciences, Nowoursynowska 159C Street, 02-787 Warsaw, Poland; grazyna_cacak_pietrzak@sggw.edu.pl; 2 Department of Grain Processing and Bakery, Prof. Wacław Dąbrowski Institute of Agricultural and Food Biotechnology—State Research Institute, Rakowiecka 36 Street, 02-532 Warsaw, Poland; agnieszka.salamon@ibprs.pl; 3 Department of Food Technology and Assessment, Division of Food Quality Assessment, Institute of Food Sciences, Warsaw University of Life Sciences, 159 Nowoursynowska St., 02-776 Warsaw, Poland; malgorzata_piecyk@sggw.edu.pl; 4 Department of Food Engineering and Process Management, Institute of Food Sciences, Warsaw University of Life Sciences, 159C Nowoursynowska St., 02-776 Warsaw, Poland

**Keywords:** oyster mushroom powder, bread, rye, chemical composition, antioxidant potential

## Abstract

This study aimed to evaluate the use of oyster mushroom (*Pleurotus ostreatus*) powder (OMP) for producing rye bread. The raw materials were low-extract rye flour and OMP, which were analyzed in terms of their nutritional and health-promoting qualities. Mixtures of rye flour with OMP were prepared, replacing 5, 7.5, and 10% rye flour with OMP. The baking quality of the tested flour samples was assessed based on their water absorption, falling number, and amylograph and swelling curve tests. The laboratory baking test was carried out using the sourdough method, prepared based on LV2 starter cultures, and the bread samples were assessed in terms of their technological, sensory, and nutritional characteristics, as well as the antioxidant potential. The OMP was characterized by a high content of basic nutrition components and a higher antioxidant potential. The addition of OMP increased the nutritional value of the rye flour and its water absorption, significantly prolonged the starch gelatinization time, and increased the xylolytic activity of the flour. The OMP enhanced the bread’s dietary fiber, minerals, protein, and phenolic compounds, and boosted its antioxidant potential. Also, the starch present in the bread with OMP was characterized by a higher pro-health value due to a higher share of slowly digestible starch. Incorporating 7.5% OMP into the rye bread formula positively affected the bread’s sensory profile in contrast to the bread with a 10% addition of OMP.

## 1. Introduction

In recent years, more and more cases of cardiovascular diseases, diabetes, overweight, and obesity have been observed around the world. Metabolic disorders are the main factors in the etiology of the above-mentioned diseases. The increased number of cases of cardiometabolic diseases and the large increase in deaths resulting from them are dependent on factors such as improper diet, the consumption of highly processed foods and stimulants, too little physical activity, and stress [1,2]. Due to the scientifically proven impact of an inappropriate diet developing lifestyle diseases, consumers are increasingly interested in functional foods and food ingredients that can benefit human health [3,4].

The growing population and ongoing climate change create the need for sustainable food production. Both of the above criteria are met by the production of oyster mushroom (*Pleurotus oyster*), which, being part of the diet, can provide the consumer with many valuable ingredients, not only nutritional ones, and its cultivation does not cause the degradation of the natural environment [5,6].

Oyster mushrooms, right after button mushrooms, are the most frequently cultivated edible mushrooms in the world. They have the ability to decompose lignocellulosic materials, making them valuable participants in breaking down organic waste and cycling nutrients in ecosystems [7]. They also are characterized by a high nutritional value and biological activity [8,9]. Oyster mushrooms are a valuable source of protein, as they contain all of the essential amino acids needed for muscle growth and tissue repair. They are particularly rich in lysine and leucine, which must be obtained from food, as the body system cannot synthesize them [10,11]. This mushroom is a rich source of vitamins, especially from the B group, which support the nervous system and energy metabolism. In addition, *Pleurotus* mushrooms also provide minerals such as potassium, phosphorus, iron, magnesium, selenium, and zinc, which are crucial for overall health and physiological processes [12]. *Pleurotus ostreatus* is rich in carbohydrates with mono- and disaccharides, which have prebiotic functions that positively influence the gut microbiome [13]. It also consists of bioactive compounds such as dietary fiber, including α- and β-glucans, and antioxidants, including flavonoids, saponins, tannins, and phenols [14,15]. The glucan content in oyster mushrooms reaches up to 9% of dry matter. The main β-glucan isolated from oyster mushrooms is pleuran [16,17]. Oyster mushrooms contain β-glucans with the (1-3),(1-6) configuration and, compared to β-glucans found in cereals with the (1-3),(1-4) configuration, show higher biological activity. The β-glucans contained in oyster mushrooms increase the activity of the immune system, causing the immune system to reduce cold symptoms, among other activities [18,19]. They also have antibacterial and anticancer properties, lower blood pressure [20,21,22], lower glucose levels [23], and have strong antioxidant properties [24,25].

Among the many food products whose recipes can be supplemented with oyster mushrooms, cereal products, which constitute the basis of the daily diet, deserve special attention [26]. The results of scientific study indicate that oyster mushrooms can be utilized in the production of wheat bread [27,28,29]. The above study showed that wheat flour enriched with powdered oyster mushrooms demonstrated significantly higher water absorption; moreover, bread with the addition of oyster mushrooms in amounts up to 10% had favorable organoleptic properties and a greater nutritional value [29]. Such bread can be classified as a product with a greater nutritional value due to the inclusion of the bioactive compounds found in oyster mushrooms. These compounds include dietary fiber, such as α- and β-glucans, as well as polyphenols, which have antioxidant potential. Moreover, it was found that the starch contained in bread enriched with oyster mushrooms has a higher share of slowly digestible starch, which results in a lower glycemic index for the bread [26,29].

According to our knowledge, no studies have examined the potential use of oyster mushrooms in the production of rye bread, which is produced by using a slightly different technology than wheat bread. The obtained research results may constitute the basis for further work toward the reformulation of rye bread to increase its health-promoting properties.

Currently, a big problem is the low activity of amylolytic and xylanolytic enzymes (responsible for the decomposition of arabinoxylans classified as non-starch polysaccharides) of produced rye flours [30,31,32]. The solution to this problem seems to be the addition of plant raw materials rich in natural enzymes, which have endoxylanase and amylolytic properties, to rye flour. The source of these enzymes is, among others, edible mushrooms, including oyster mushrooms [33,34]. Therefore, it was justified to undertake a study to check whether adding oyster mushrooms to rye flour will increase its amylolytic and xylanolytic activity, and thus improve the baking properties of the rye flour and the overall quality of the obtained rye bread.

This study aimed to evaluate how oyster mushroom powder affects the baking quality of rye flour, and the physicochemical, nutritional, and sensory qualities, as well as the pro-health potential, of rye bread.

## 2. Materials and Methods

### 2.1. Materials

The raw materials tested included low-extract rye flour, sourced from a reputable industrial mill in Poland, as well as freeze-dried oyster mushrooms. The oyster mushrooms were purchased from a Polish market. To prepare the mushrooms for freeze-drying, they were first sliced into small pieces, which were placed in a GN 50Vd26 freezer (Liebherr, Bulle, Switzerland), and then subjected to a deep freeze at a temperature of −32 °C. The mushrooms underwent a thorough freeze-drying process using an Alpha 1–4 LSCplus freeze-dryer (Martin Christ Gefriertrocknungsanlagen GmbH, Osterode am Harz, Germany). To receive the oyster mushroom powder, the obtained mushroom lyophilizate was milled by a laboratory mill (Sadkiewicz Instruments, Bydgoszcz, Poland) to achieve a fine powder, with practice sizes of less than 0.5 mm. The oyster mushroom powder was then stored at room temperature for use.

Concurrently, the rye dough was prepared using a commercial Livendo^TM^ LV2 sourdough starter and compressed baker’s yeast (Lesaffre Group Company, Warsaw, Poland). To enhance the flavor and texture of the dough, salt was also incorporated.

### 2.2. Primary Chemical Composition and Health-Promoting Properties of Raw Materials and Tested Bread

The contents of the primary chemical components (i.e., the moisture, protein, ash, fat, dietary fiber, and carbohydrate contents) in the raw materials, i.e., the rye flour (RF) and oyster mushroom powder (OMP), and the obtained bread samples were determined according to the methodology described by Salamon et al. [35]. The cereal β-glucan content (β-Glc) in the RF was determined with the methodology outlined by Salamon et al. [36]. This enzymatic procedure is specific for mixed-linkage (1-3),(1-4)-β-D-glucan found in cereal grains, milling fractions, and other food products. In turn, the fungal β-glucan (β-Glf) and α-glucan contents (α-Gl) in the OMP were determined by the spectrophotometric method (DU-530 spectrophotometer; Beckman, Wycombe, UK) using the enzymatic Yeast and Mushrooms β-Glucan Assay Kit (Neogen, Bray, Co., Wicklow, Ireland) with the procedure described in Assay Protocol 08/23 [37]. The method is dedicated to determining the (1-3),(1-6)-β-D-glucan present in mushrooms and algae, fungi, and yeast. Due to the limitations of the enzymatic method for determining the fungal β-glucan and α-glucan contents in bread related to the presence of starch, it was not possible to determine the content of these components in the evaluated bread. All of the results from these tests were reported as percentages of dry matter (d.m.).

The content of the total polyphenol (TP) and antioxidant activity using the DPPH method in the used raw materials and the tested bread samples were determined according to the methodology by Szczepańska et al. [38]. The results for the TP content, as mg of gallic acid equivalent (GAE), and the antioxidant activity (DPPH), as µM Trolox, were expressed per one gram of dry matter (d.m.).

### 2.3. Baking Quality of Tested Rye Flour and Its Mixtures with Oyster Mushroom Powder

The baking quality of the rye flour (RF) and its mixtures with 5, 7.5, and 10% of oyster mushroom powder (OMP) was determined based on the following analyses: the water absorption, falling number, amylograph characteristics, and swelling curve test. The falling number was determined using an FN 1500 device, manufactured by Perten Instruments in Hägersten, Sweden, according to ISO 3093 [39]. To assess the water absorption of the flour samples, a Mixolab device from Chopin Technologies (Villeneuve-la-Garenne, France) was employed. This assessment was conducted according to ISO 17718 [40]. The amylograph test was determined using an amylograph from Brabender (Duisburg, Germany) according to the procedures detailed in ISO 7973 [41]; finally, the swelling curve test was conducted with the methodology described in detail by Stępniewska et al. [42].

### 2.4. Procedure of Rye Baking Trial

The rye loaves of bread were made with sourdough (SD), which was prepared from 30% of flour used in baking, the water needed to obtain a yield of 250%, and starter (SAF Levain LV2 from Lesaffre) in an amount of 1% in relation to the flour used in the SD. The SD fermentation occurred over a period of 20 h, maintained at a controlled temperature of 30 °C and a relative humidity of 75%. After this time, the SD was mixed with the remaining amount of flour, constituting 70% of the flour specified in the baking recipe and water (based on the absorption determined by Mixolab and adjusted by an extra 4% to ensure adequate hydration). The formulation also included 2% of baker’s yeast and 1.7% salt. The total amount of water added to the dough was reduced by the amount used to SD. The next step of the dough preparation and baking procedure was made according to the methodology described in detail by Stępniewska et al. [31]. After baking, the bread samples were carefully removed from the molds and allowed to cool at room temperature. After being cooled, the loaves of bread were packed in polyethylene bags to preserve their freshness. Next, the bread was stored for 72 h at room temperature (22 ± 2 °C).

### 2.5. Technological Properties of Bread

Twenty-four hours after baking, the bread loaves were weighed. In addition to the weight measurement, several key parameters were determined. This assessment included measuring the loaf volume, the bread crumb moisture content, and hardness, which were determined using the methodology outlined by Stępniewska et al. [42]. The hardness of the bread crumb was also determined seventy-two hours after baking so to define the increased hardness of the bread crumb during the storage of the bread.

### 2.6. Color Coordinates of Bread Crumb

The parameters describing the color of the bread crumb included the L* (lightness), a* (redness), b* (yellowness), as well as C* (chroma). These measurements were performed in accordance with the methodology established by Salamon et al. [35]. Parameter ΔE, which is the absolute color difference between the control sample of the bread (CB) and the bread samples with the addition of the OMP, was calculated based on the formula presented by Ignaczak et al. [43].

### 2.7. Energy Value of Bread

The designation of the basic components that shape the nutritional value of the bread samples is given in Section 2.2. To estimate the energy value (EV) of each bread sample in kilocalories per 100 g (kcal/100 g), we used the formula given by Salamon et al. [44]. The results for the protein (P_B_), carbohydrate (C_B_), fat (F_B_), and total dietary fiber (TDF_B_) contents, given as percentages of dry matter (d.m.), were converted to percentages.

### 2.8. Total Starch Content and Its Digestibility

The total starch content was determined by using a total starch assay kit (Megazyme, Bray, Co., Wicklow, Ireland). In vitro starch digestibility was conducted according to Englyst et al. [45] with some modifications, as described previously [46]. Briefly, the starch was digested with pancreatin from porcine pancreas (P-7545, activity 8 × USP/g; Sigma, St. Louis, MO, USA) and amyloglucosidase (EC 3.2.1.3., 3300 U/mL; Megazyme, Bray, Co., Wicklow, Ireland). Starch classifications based on the rate of hydrolysis included rapidly digestible starch (RDS; digested within 20 min), slowly digestible starch (SDS; digested between 20 and 120 min), and resistant starch (RS; undigested after 120 min). All of the starch values obtained for the bread were corrected for free glucose. The amounts of glucose were measured by the glucose oxidase–peroxidase assay kit (GOPOD, K-GLUC, Megazyme, Bray, Co. Wicklow, Ireland).

### 2.9. Sensory Evaluation of the Tested Loaves of Bread

The sensory assessment of the tested loaves of bread was conducted 24 h after baking. The evaluation panel included 18 trained assessors. They included students who had passed the relevant subjects and practical classes, and employees of WULS (Warsaw University of Life Sciences) who had repeatedly performed similar assessments of bakery products. Participants were chosen based on their self-reported good health, the absence of gluten allergies, and the regular consumption of bread. The bread samples were cut into 2 cm thick pieces, coded, and presented in a random order to ensure an unbiased evaluation. Based on the 9-point hedonic scale, the tested loaves of bread were assessed, where 1 indicated very undesirable and 9 represented very desirable. The quality attributes that were assessed as part of the sensory evaluation were the crumb color and texture, taste, aroma, and overall acceptability of the bread. The evaluation card contained the following explanations of the individual sensory characteristics of the bread:

- Crumb color: please assess the visual attractiveness of the bread crumb color, with particular emphasis on its saturation and uniformity;
- Crumb texture: please rate the crumb texture of the bread, focusing on its softness, elasticity, and mouthfeel; please take into account how the crumb is chewed and how it feels on the palate;
- Taste: please assess the balance between the sensation of sweet and salty, and pay attention to the presence of any foreign flavors; if you notice any foreign flavors, please specify them;
- Aroma: please assess the intensity and complexity of odors and pay attention to the presence of any foreign odors; if you notice any foreign odors, please specify them;
- Overall acceptability: considering all of the above sensory characteristics, please assess the overall desirability of the tested bread sample.

### 2.10. Statistical Analysis

The data collected during our study were subjected to a thorough analysis using Statistica 13 software, developed by StatSoft in Cracow, Poland. All of the measurements were performed for at least three repetitions. A one-factor analysis of variance (ANOVA) was utilized, followed by Tukey’s test, which allowed us to identify which groups were significantly different from one another. The statistical tests were performed with a significance level set at α = 0.05. Also, a principal component analysis (PCA) was performed. For the PCA analysis, the average values for each parameter measured in the flour and bread samples were used, which aligned well with the analysis performed on all of the repetitions.

## 3. Results and Discussion

### 3.1. Chemical Characteristics of Raw Materials of Baking

Table 1 presents the results of the basic chemical composition of the rye flour (RF) and oyster mushroom powder (OMP) used to prepare the baking mixes. The conducted studies showed significant differences between the assessed samples of the RF and OMP in terms of the basic compounds, and the nutritional and health-promoting values (Table 1). The quality parameters, i.e., the moisture, ash, and protein contents of the RF samples used in the research, were appropriate for low-extract rye flours and were consistent with the authors’ previous results [32]. Our results showed that the OMP samples were rich in protein, total dietary fiber, ash, and fat contents but lower in carbohydrate content than those of the RF. The OMP sample was characterized by an approx. 40% lower moisture content, indicating that the OMP could be safely stored. The OMP sample had higher protein (approx. 3.2 times), ash (approx. 9.5 times), and fat (approx. 1.4 times) contents than the RF sample. When assessing the carbohydrate content in the bakery raw materials, it was found that the OMP sample was characterized by more than three times lower levels of this component compared to the RF sample (on average, 25.08 and 82.40% d.m., respectively). Other authors, who added oyster mushrooms to produce wheat shortbread biscuits, obtained similar relationships [47,48,49]. Agarwal et al. [50] reported that *Pleurotus* mushrooms contained 40.6–53.3% carbohydrates, with these contents including fiber, which consists of structural polysaccharides, i.e., β-glucans, chitin, hemicelluloses, and pectic substances. In oyster mushrooms, the authors showed the presence of sugars, such as glucose, fructose, mannitol, trehalose, myo-inositol, and sucrose. In our research, the higher TDF content was determined in the OMP sample (on average, 44.19% d.m.) compared to the RF sample (on average, 8.91% d.m.), with the fraction of insoluble dietary fiber (IN-DF) being present in larger amounts in both samples. Almost 60% of the TDF content was the fraction of insoluble dietary fiber (IN-DF) contained in the RF sample, while, in the OMP sample, this fraction constituted approx. 90% of the TDF content. The study conducted by Uriarte-Frías et al. [49] indicated that the crude fiber content in natural oyster mushroom flour was about five times higher than in whole-wheat flour, and amounted to an average of 10.37 and 1.99%, respectively.

The polysaccharides found in cereal grains and mushrooms include glucans, which are also part of the dietary fiber fraction [51,52]. Oyster mushrooms contain fungal β-glucans, occurring in the (1-3),(1-6)-type configuration, which, compared to cereal β-glucans that typically have (1-3),(1-4) linkages, show a higher biological activity [14]. The content of fungal β-glucans in the OMP sample was found to be considerably higher than that of the rye flour (on average, 42.08 and 1.48% d.m., respectively). Vetter [51] reported that the content of fungal β-glucan, determined by Megazyme’s enzymatic method in oyster mushrooms, *Pleurotus ostreatus*, was similar to our result, and amounted to an average of 40.34% d.m.

The RF was characterized by a lower total polyphenol content (TP) (on average, 0.68 mg GAE/g d.m.) and exhibited reduced antioxidant capabilities, quantified as 0.95 µM Trolox/g d.m., compared to the OMP, which contained more than seven times higher amounts of TP and more than eight times higher antioxidant activity (DPPH) than the tested RF. Morris-Quevedo et al. [48] stated that oyster mushroom powder exerted about 10 times higher scavenging activity against the DPPH radical than the control high-extract wheat flour. Kozarski et al. [53] reported that bioactive compounds responsible for the antioxidant properties of oyster mushrooms, such as polysaccharides, polyphenols, vitamins, minerals, and carotenoids, might vary depending on factors including the specific strains used, the substrates, the method of cultivation, and the overall growth conditions.

### 3.2. Baking Quality of Rye Flour and Its Mixtures with Oyster Mushroom Powder

The baking quality of the flour samples was assessed using parameters such as the falling number value, water absorption, amylograph, and swelling curves (Table 2). The falling number for the rye flour and its mixtures with the OMP was measured to be above 200 s, which indicates that all of the flour samples exhibited low levels of α-amylase activity [31]. It was found that adding OMP to the rye flour significantly lowered the falling number value from 236 s for the rye flour (RF) to 212 s for the flour enriched with 10% of OMP (RF10). However, the found differences did not exceed the permissible limits of reproducibility specified in ISO 3093 [39] for falling numbers and, therefore, are not significant from a technological point of view.

The addition of OMP into the rye flour increased the water absorption (WA) of the tested flour from 61.3% for the RF to 65.1% for the RF10. Probably, the increase in WA was caused by the dietary fiber and protein content introduced from the OMP, which keeps more water [54]. Further support for this finding can be found in the study conducted by Biao et al. [55], who added oyster mushroom powder to wheat flour. In these studies, enriching wheat flour with OMP at the level of 10% resulted in an increase in WA by 2.7 percentage points.

The study showed that the OMP has a significant impact on the swelling curve parameters. The parameter VA, which indicates the initial viscosity at 30 °C, reflects the capacity of rye flour ingredients to absorb water and swell immediately after preparing the suspension, and was increased from 105 AU for the RF to 270 AU for the RF10. In our opinion, the protein and dietary fiber contained in the OMP caused a significant increase in the VA. The study showed that the viscosity when the slurry reached 42 °C (VB) for the RF5 flour was twice as high compared to the control flour (with average values of 200 and 105 AU, respectively). An increase in the OMP addition to 7.5% further increased the VB to 270 AU. The study showed that the VB for the slurry made from the RF10 flour was at the same level with respect to the VB for the RF7.5 flour. The final viscosity (VC) also saw an increase, rising from 100 AU for the control flour to 235 AU with respect to the RF7.5 flour. Increasing the OMP addition to 10% resulted in a reduction in the VC to 200 AU. It was noticed that the viscosity increased only with respect to the RF7.5 flour during its heating from 30 to 42 °C. This was probably caused by a higher amount of swollen substances and the increased ability to swell and bind water, as well as the degree of their enzymatic decomposition, in these flour samples [42]. Based on the value of the logarithmic decrease in the viscosity of the suspension after its 30-min hold at 42 °C (Log), we revealed that the enriched rye flour into the OMP caused a significant increase in the activity of the enzymes that decompose hemicellulose, mainly arabinoxylans, with respect to the rye flour. This allows us to conclude that the OMP used in this study was a source of naturally occurring enzymes that degrade hemicelluloses. The addition of OMP into the rye flour caused a significant increase in the Log from 22 for the rye flour to 130 concerning the RF10 flour. The hemicellulose hydrolyzing enzymes, mainly xylanases that decomposed arabinoxylans in the rye flour, firstly improved the dough’s physical properties, making it easier to process, and, secondly, improved the bread quality by positively affecting its volume and crumb structure, and extending the freshness of the bread [56]. With an optimal xylanase activity, the elasticity of the dough increases, affecting the ability of the dough to retain gases and increasing the volume of the bread loaf. Excessive xylanase activity may hinder gas pretention during fermentation, negatively impacting the bread volume [42].

All of the flour samples tested in this study were characterized by an amylograph maximum peak viscosity (V_max_) at the optimal level (in the range from 400 to 600 AU), which is necessary for high-quality rye bread production [32]. The addition of the OMP significantly reduced the V_max_, decreasing from 520 AU for the RF to 450 AU for the RF10 flour. Liu et al. [57] established a significant relationship between the relative starch content in the tested material and the V_max_. The cited studies showed that increasing the dietary fiber content from wheat bran correlated with a decrease in the relative starch content in the sample, leading to a reduction in the V_max_. Considering the above results, it can be assumed that the slurry obtained from the tested rye flour enriched with OMP in our study was characterized by a lower V_max_ because the OMP lowered the relative starch content in the tested material. It was found that the RY was characterized by the highest initial temperature of starch gelatinization (T_I_) (on average, 55.0 °C) compared to all of the flour samples enriched with the OMP. The rye flour enriched with 5 and 7.5% OMP (RF5 and RF7.5, respectively) was characterized by a slightly lower T_I_ (on average, 54.5 °C), while the T_I_ for the RF10 flour was 53.5 °C, and, as the statistical analysis showed, was significantly lower compared to the RF0 flour. In the case of the final temperature of the starch gelatinization (T_F_), it was shown that the OMP significantly increased the T_F_. The RF was characterized by the significantly lowest T_F_ (on average, 72.0 °C) compared to the rye flour mixtures with OMP. The study showed that the T_F_ for the RF5 flour was as much as 14.0 °C higher than for the RF, the average value of which was 86.0 °C. The RF7.5 and RF10 flours were characterized by a slightly higher T_I_ value, averaging 88.0 and 89.5 °C, respectively. Similar findings were reported by Biao et al. [55], who added oyster mushrooms to wheat flour in amounts ranging from 5 to 25%. In the above studies, the T_F_ for the wheat flour with the addition of 5% mushroom powder was 16 °C higher than the control flour. Further, an increase in the addition of mushrooms did not cause significant changes in the T_F_. Ng et al. [58], who examined the effect of adding *Pleurotus sajorcaju* powder to wheat flour, stated that the polysaccharides contained in mushrooms constitute a physical barrier to starch-degrading enzymes and inhibit the starch gelatinization process, whereas, in the opinion of Tu et al. [59] and Olawuyi and Lee [60], the proteins present in mushrooms delay the starch gelatinization process. These components, like polysaccharides, also compete with starch for water absorption, extending the gelatinization process. Based on the above research, we suppose that the significant increase in the starch gelatinization temperatures (T_F_) in the flour samples with the addition of OMP may be caused by the competition of starch with proteins and the dietary fiber contained in the OMP in water absorption, which limited the swelling of starch and increased the T_F_ [59,60].

### 3.3. Physicochemical Properties of Rye and Enriched Bread Samples

The findings of the physicochemical properties of the bread samples, comprising the control bread (CB) and the enriched variants with the OMP at concentrations of 5, 7.5, and 10%, designated as OMB5, OMB7.5, and OMB10, respectively, are presented in Table 3. The available literature concerning the addition of mushrooms in wheat flour shows that the supplementation of wheat flour with mushroom powder has a negative effect on the wheat bread volume [27,29,54]. This is caused by the weakening of the gluten network, which is crucial for retaining gases during the fermentation and baking processes. This weakening leads to a diminished ability of the dough to trap carbon dioxide, resulting not only in a reduction in the volume of the bread and an increase in the crumb hardness but also in the deformation of the bread’s shape [61,62]. Our study showed that the supplementation of the rye flour with OMP at 5 and 7.5% levels did not significantly affect the volume of the obtained loaves of bread. The loaves of bread made from rye flour with 5 and 7.5% OMP had a similar volume to the control bread, i.e., an average of 195 cm^3^/100 g for the CB and OMB5, as well as 193 cm^3^/100 g for the OMB7.5 bread. Probably because no gluten network was formed during the production of the rye bread, as in the case of the wheat dough, there was no negative effect of the oyster mushroom powder on the volume of the rye bread. Our study showed that only the bread made from the flour enriched with OMP at the level of 10% was characterized by a significantly lower volume (on average, 185 cm^3^/100 g). We noticed that this bread, compared to the other loaves of tested bread, was characterized by the highest content of the insoluble fiber fraction and the highest share in the total fiber content (Table 4), and, compared to the OMB7.5 bread, it was described by the same soluble fiber content, with a 124% higher insoluble fiber content. This may indicate that, during the baking of bread from the RF10 flour, the insoluble fraction of dietary fiber was transformed into soluble forms to a much lower extent, negatively affecting the bread’s volume and its crumb structure [63]. Probably during the baking of the bread from the RF10 flour, the insoluble hemicelluloses contained in the dietary fiber formed complexes with proteins during the rye dough production, hindering protein denaturation, which led to the lack of free water in the starch gelatinization process, affecting a decrease in the gas retention capacity, which reduces the volume of bread and increases its hardness. Also important, with respect to rye dough, are the mutual interactions between the rye flour proteins, which undergo cross-linking during the influence of proteolytic enzymes [64]. Therefore, we assume that the OMP used in our studies was probably a source of proteolytic enzymes that caused protein cross-linking, creating a protein network that prevented a reduction in the volume of bread made from the RF5 and RF7.5 flours.

The rye bread samples tested in our study showed significant differences in the bread crumb moisture (M_B_). The control rye bread (CB) had a notably lower M_B_, averaging 46.7%. When OMP was added to the rye flour, there was a significant increase in the M_B_. The above parameter for the rye bread enriched with OMP ranged from 47.7% to 48.8% for the OMB5 and OMB10, respectively. A similar result was observed by Ndung et al. [28], who examined the effect of oyster mushroom powder on various quality parameters of wheat bread. The above studies demonstrate that the M_B_ for wheat bread supplementation with 5 and 10% OMP compared to the control bread was higher by 4.4 and 4.5%, respectively. In studies conducted by Okafar et al. [27], when wheat flour was replaced with mushroom powder at a level from 5 to 25%, the obtained bread samples were characterized by an M_B_ in the range from 31.8 to 32.6%, but the differences were statistically insignificant. Schleißinger et al. [65] described that dietary fiber, due to a higher than starch water-holding capacity, has a significant impact on the bread crumb moisture. Considering the above contention, the higher M_B_ for the tested bread samples enriched with OMP in our studies was caused by the dietary fiber introduced with mushrooms.

The hardness of the bread crumb 24 h after baking (H_24_) for the OMB5 was at a similar level to the CB (averages of 39.1 and 39.4 N, respectively), but, 72 h after baking, the bread crumb hardness (H_72_) for the OMB5 was at a significantly lower level than for the CB (averages of 56.9 and 68.7 N, respectively). From all of the tested bread samples, the lowest H_24_ and H_72_ were characterized by the OMB7.5 bread, in which case, the values of the discussed parameters were nearly 14 and 23% lower compared to the CB (on average, 34.0 and 53.0 N, respectively). The OMB10 bread had significantly higher H_24_ and H_72_ values, averaging 58.6 and 78.5 N, respectively. Compared to the control bread (CB), the H_24_ and H_72_ for the OMB10 were 49 and 25% higher, respectively. In contrast to our results, Sławińska et al. [61], who studied the impact of the supplementation wheat flour with *Aspericus bisporus* powder on the quality parameters of wheat bread, revealed that the mushrooms increased the bread crumb hardness even at the lowest level of supplementation. In the opinion of the authors, the increase in the bread crumb hardness is related to damaging the gluten matrix in the wheat dough by the chemical substances present in the mushrooms. Also, Gaglio et al. [66] showed that the incorporation of *Pleurotus energii* mushroom powder into wheat flour significantly increased the hardness of the bread crumb. In the above studies, the crumb hardness of the bread with 10% mushroom powder was 66% higher, measuring 28.7 N, compared to the CB bread, which averaged 17.2 N.

Among the tested rye bread samples analyzed in our study, the control bread (CB) exhibited the highest increase in the hardness of the bread crumb during two days of storage (HI) (on average, 29.3 N). The HI for the bread samples enriched with OMP ranged from 17.8 to 19.9 N for the OMB5 and OMB10, respectively. The differences found in the HI in relation to the bread samples with the OMP were statistically insignificant. Probably, the anti-staling properties of the bread crumb samples enriched with OMP may result, firstly, from the higher content of dietary fiber, which can bind water and prevent water loss during storage, and, secondly, from the interactions with starch, delaying its retrogradation process, which is one of the reasons for bread staling [65,67].

The color of a food product, including bread, is an important element in assessing consumer acceptability. Utilizing the CIA L*a*b* system provides more possibilities for color characteristics in bread samples containing oyster mushroom powder. The lightness of the color of the crumb bread supplemented with OMP, determined by the L* parameter, darkened with the increase in the additive. Namely, the highest value of the L* parameter was found in the CB bread (on average, 56.3), whereas the loaves of bread fortified with 7.5 and 10% OMP powder values were the lowest (on average, 48.9 and 48.3, respectively) and did not differ significantly from each other (Table 3). Moreover, increasing the addition of OMP to the rye bread resulted in notable increases in the other color parameters, including a*, b*, and C*. The a* parameter was in the range of the redness color, on average, from 3.66 to 6.01 (for the CB and OMB10 bread, respectively). However, the b* parameter values were in the range of the yellow color, i.e., averages from 17.00 (for CB) to 20.12 (for the OMB7.5 and OMB10). Similar trends for the change in the color parameter values were observed by Losoya-Sifuentes et al. [29] for wheat bread with different supplementations of OMP, ranging from 0 to 20%. The occurrence of red and yellow pigments in the bread crust and crumb with the addition of OMP may be related to the so-called non-enzymatic browning reactions that occur between amino acids and sugars under the influence of high temperatures during bread baking. However, Zhang et al. [68] reported that the change in the crumb color may not be related to the baking temperature, as is the case with the bread crust, but rather due to the addition of mushrooms. In addition, in our study, the bread samples with the added OMP showed significantly higher chroma (C* parameter) values than the control rye bread (CB), the value of which changed from 17.39 to 21.00 (on average for the CB and OMB10 bread samples, respectively). Considering the values of the lightness and saturation of the bread crumb color (Table 3), the color of the bread crumb samples can be described as slightly grayish (for the CB) to grayish and muted slightly darker (for the OMB10). In general, the bread samples with the 5 and 7.5% OMP addition did not differ (*p* > 0.05) in the color parameters. The color difference, denoted as ΔE, ranged between 6.89 for the OMB5 and 8.87 for the OMB10. According to the criteria given by Karma [69], the value of ΔE indicates that these differences are noticeable to the human eye.

### 3.4. Basic Chemical Composition of Tested Bread Samples

Table 4 details the results concerning the basic ingredients of the bread that influence its nutritional and caloric values. The ash content in bread indicates the presence of mineral compounds. In the obtained bread samples, replacing part of the rye flour (RF) with OMP significantly increased the ash level in the bread, even by approx. 30%, in the samples with 7.5 and 10% OMP additions compared to the control sample (CB). Our study showed statistically significant differences (*p* < 0.05) between the CB bread and the samples supplemented with OMP regarding the content of various components that directly influence the caloric value (EV) of the food products. As the amount of oyster mushrooms incorporated into the rye bread increased, we observed a rise in the level of protein, fat, and dietary fiber, coupled with a decrease in carbohydrates and starch in the finished product. These findings are similar to previous research [29,47,48], where wheat flour was replaced with oyster mushroom powder in bread. The protein content in rye bread samples with OMP ranged from 7.53% d.m. (CB) to 9.31% d.m. (OMB10%). When compared to the CB bread, the addition of OMP caused an average protein increase of approx. 12%, while the studied bread samples were statistically significantly different (*p* < 0.05) in terms of the protein content. The analyzed bread samples contained small amounts of fat (ranging from 0.05% d.m. for the CB to 0.13% d.m. for the OMB10). The starch content of the CB was at the level of 72.75% d.m. and was consistent with that reported in the literature, at 61.1–77.4% d.m. [70,71]. In the bread samples with OMP, the starch content decreased from 70.24% d.m. (OMB5) to 66.49% d.m. (OMB10) with an increasing OMP addition. This difference can be attributed to the significantly lower starch content of the OMP compared to the rye flour [72]. The observed reduction in the carbohydrate content in the bread samples (on average, from 2.21 to 5.62% d.m. in the OM5 and OM10 samples, respectively) resulted mainly from replacing part of the rye flour with OMP as a source of dietary fiber, including β-glucans, and protein (Table 4), and from the transformation of the starch and sugars during dough fermentation with the participation of lactic acid bacteria and yeast. On the other hand, the TDF content in the bread samples ranged on average from 9.43 to 12.82% d.m. for the CB and OMB10, respectively. No significant differences in the soluble dietary fiber (S-DF) content were found between the bread samples with the OMP added (on average, from 4.26 to 4.47% d.m. in the OMB5 and OMB10, respectively), although significantly lower S-DF was present in the control bread (CB) (on average, 3.66% d.m.). In the tested rye bread sample with OMP, the share of the insoluble dietary fiber (IN-DF) fraction constituted approx. 60-65% of the TDF content. No significant difference in the IN-DF was found between the CB and OMB5 bread samples (on average, 6.11 and 5.77% d.m., respectively). The OMB7.5 and OMB10 bread samples, compared to the OMB5 bread, were characterized by a 10 and 36% higher IN-DF (on average, 6.73 and 8.35% d.m., respectively). Due to the fact that the tested rye bread samples enriched with oyster mushrooms contain from 5.03 to 6.57% of dietary fiber (Table 4), according to the guidelines of European Union (EU) Regulation No. 1924/2006 [73], the CB, OMB5, and OMB7.5 bread samples can use nutrition claims such as “source of fiber”, as their fiber content is above 3 g per 100 g, while, for the OMB10 bread, nutrition claims such as “source of high fiber” are possible due to a fiber content above 6 g per 100 g for the bread in this sample.

For the tested samples of rye bread, the estimated energy value (EV) ranged from 207 kcal per 100 g to 215 kcal per 100 g (for the OM10 and CB, respectively) (Table 4). Replacing part of the rye flour with OMP in the recipe significantly reduced the EV of the bread samples. Specifically, an average reduction of 5 kcal per 100 g was observed with a 10% substitution of whole-wheat flour with the addition of OMP in the bread recipe, as reported by Morris-Quevedo et al. [48]. Also, in the study by Sławińska et al. [47], similar relationships were obtained for shortbread cookies enriched with *Pleurotus ostreatus* powder.

The content of total polyphenol (TP_B_) and the antioxidant activity (DPPH_B_) in the rye bread samples enriched with OMP are presented in Table 4. Supplementing the rye flour (RF) with OMP caused an increase (from approx. one-and-a-half to two times) in the TP_B_ in the bread samples, i.e., from 0.74 mg GAE/g d.m. (CB) to 1.14 and 1.64 mg GAE/g d.m. (for the OMB5 to OMB10, respectively). A similar trend was observed in the case of the antioxidant activity of the tested bread samples. Compared to the CB, the addition of OMP contributed to the increase in the DPPH radical quenching capacity by approx. 50–80% by the antioxidants in the bread samples enriched with the OMP. Many studies [48,49,74,75] demonstrated the existence of a relationship between the antioxidant capacity and the amount of polyphenols in the studied cereals, pseudocereals, legumes, and their products. According to Sławińska et al. [47], the increase in the TP_B_ content in bread samples with OMP could be due to the dietary fiber and polysaccharides found in mushrooms. These components may limit the release of bound polyphenols. Meanwhile, Han and Koh [76] reported that the content of phenolic compounds changed during dough mixing, fermentation, and baking, but the baking process contributed to a decrease in the level of polyphenols (by about 20–26%) due to the high temperatures involved. In addition, the increased antioxidant activity (DPPH_B_) in the bread enhanced with OMP can be attributed, on the one hand, to a higher TP content in the OMP than in the rye flour and the presence of other antioxidants, such as melanoidins, formed by the Maillard reaction during the breadmaking [47,75].

### 3.5. In Vitro Starch Digestibility

In the control bread, the proportion of RDS, SDS, and RS was found to be 83.07%, 4.19%, and 12.74%, respectively (Table 5). These values are consistent with the data reported in the existing literature [77]. In general, the digestibility of starch in rye products is lower than that in wheat products [78]. This is due to the fact that the fiber content of rye flour is higher than that of wheat flour. The soluble dietary fiber present in rye products, particularly arabinoxylans, increase the viscosity of the digesta, making it more difficult for amylolytic enzymes to access the starch [32,78].

Incorporating OMP into the rye flour reduced the RDS proportion to a similar level (~79%) across all of the enriched bread variants, with no statistically significant differences observed. Consequently, the sum of the SDS and RS increased in all of the bread variants with the OMP. The proportion of SDS was significantly higher in both the OMB5% and OMB7.5% bread samples than in the control bread. Furthermore, the proportion of RS was significantly higher in the OMB10% bread than in the CB. Studies reported in the literature have also observed a reduction in the digestibility of cereal products with the addition of oyster mushrooms, although it should be noted that most of these studies focused on wheat products. For instance, the incorporation of mushrooms led to a reduced rate of starch hydrolysis in wheat bread samples [62], whereas, in biscuits, there was an observed increase in the SDS and RS contents [58]. The alterations observed can be attributed to the rise in dietary fiber, polyphenols, and protein content in bread incorporating OMP (Table 4), as well as the interactions between these components and starch. Studies in the literature have shown that interactions occurred between non-starch polysaccharides and starch, resulting from the formation of non-covalent bonds between these components. Furthermore, long-chain non-starch polysaccharides and proteins surrounded most of the starch granules [59]. These factors affect the functional properties of starch. For instance, the encapsulation of starch granules by fibers and proteins inhibits the swelling of the granules, which, consequently, do not gelatinize completely in hydrothermal processes [75]. Native rye starch is a type A starch that, in its non-gelatinized state, contains a high proportion of SDS [79]. This may explain the increase in the proportion of this fraction in the OMB5% and OMB7.5% bread samples compared to the CB. In addition, an increase in the viscosity of the digesta has been observed, limiting enzyme access to the starch [59]. The starch digestibility may also be affected by the presence of mushroom polyphenols. A negative correlation was found between the polyphenol content and the glycemic index (GI) as determined in vitro in mushroom-enriched extrudates and noodles [80]. Polyphenols have the capacity to bind to starch, forming either inclusion or non-inclusion complexes. Additionally, they have the ability to inhibit the activity of both α-amylase and α-glucosidases [81]. The formation of stable starch–polyphenol complexes may occur as a consequence of the type of polyphenols involved, leading to a rise in the RS content [82]. The highest concentrations of polyphenols and fiber were observed in the OMB10% sample (Table 4), which may have contributed to the conversion of SDS into RS.

### 3.6. Sensory Evaluation of Tested Bread Samples

**Figure 1:**
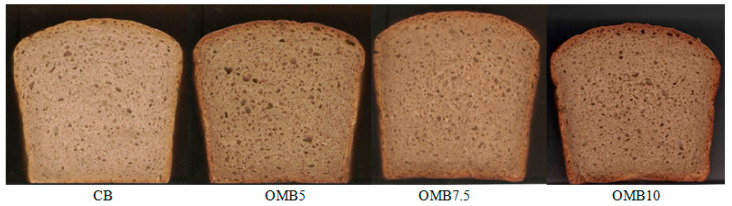
Picture of the control and enriched bread samples: CB—control bread; OMB5, OMB7.5, and OMB10—bread enriched with 5, 7.5, and 10% of oyster mushroom powder, respectively.

The pictures of the control and enriched bread loaves are presented in Figure 1, while the sensory evaluation results of the tested bread samples are presented in Table 6.

It was found that the control bread (CB) was characterized by a much lighter color in the crumb compared to the bread made from rye flour supplemented with oyster mushroom powder. The darkening of the color was evident in the case of the bread crumb with 10% OMP, which was rated as significantly lower in terms of the color (an average of 5.8 points) than the CB (an average of 7.5 points), as well as for the OMB5 and OMB7.5 (averages of 7.7 and 7.6 points, respectively). Regarding the smell, the evaluators highly accepted the bread with 7.5% and 5% OMP (averages of 8.5 and 8.2 points, respectively), and the CB was also rated highly (an average of 7.6 points). A significantly lower score for smell (4.5 points) was given to the bread with 10% OMP, which, according to the evaluators, had too much of a mushroom smell. For the taste, the bread with 7.5% OMP received the highest rating, averaging 8.6 points, followed closely by the 5% OMP bread, with an average of 8.1 points. The evaluators indicated that the 7.5% OMP bread had a significantly better taste than the CB, with an average score of 7.5 points. In contrast, the bread containing 10% OMP received the lowest taste score of 2.5 points, as the evaluators detected a strong and unpleasant bitter aftertaste. Similarly, in the research conducted by Ndung et al. [28], the taste of wheat bread enriched with 10% mushroom powder was not accepted by the evaluators due to the noticeable bitter aftertaste, described as meaty by a member of the evaluation panel. Regarding the texture, the CB, OMB5, and OMB7.5 were rated at a similar level (averages from 8.0 to 8.3 points), while the OMB10 was rated significantly lower (5.3 points) due to its more compact and harder crumb than the other samples. Summarizing the sensory evaluation results, it was found that bread with 7.5 and 5% OMP had the highest overall acceptability (OA) (averages of 8.1 and 8.0 points, respectively). In comparison, the bread with 10% OMP had the lowest acceptance (an average of 3.5 points). In the research conducted by Ndung et al. [28], wheat bread with 10% dried mushrooms also obtained a low OA, which indicates that, at this level of addition, there is a significant deterioration of the sensory properties of wheat bread.

### 3.7. Principal Component Analysis (PCA)

**Figure 2:**
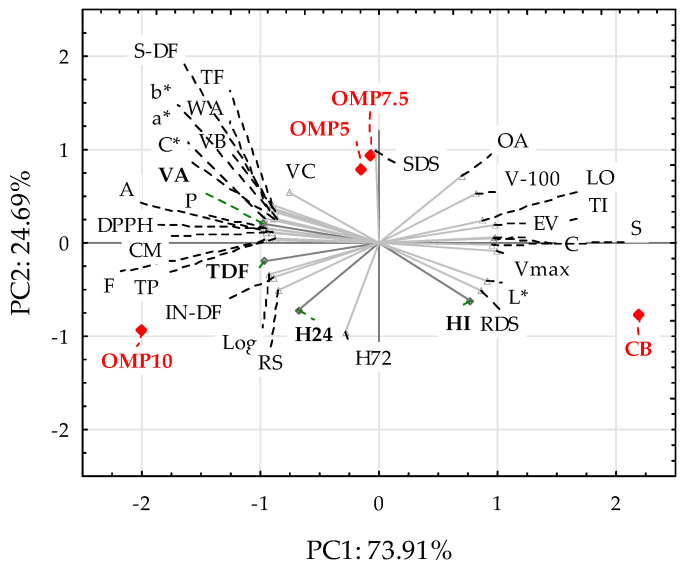
Biplot of the PCA score plot and principal component loading plots, PC1 and PC2, with located bread samples marked in red. Explanations: Vmax—amylograph maximum peak viscosity; TI and TF—initial and final temperature of the starch gelatinization, respectively; LO—falling number; WA—water absorption; VA—initial viscosity at 30 °C; VB—viscosity when the slurry reached 42 °C; VC—final viscosity after holding a slurry for 30 min at 42 °C; Log—logarithmic decrease in the viscosity at 42 °C; RDS and SDS—rapid and slowly digestible starch, respectively; RS—resistant starch; P, A, F, and C—protein, ash, fat, and carbohydrate contents in the bread, respectively; TDF, IN-DF, and S-DF– total, insoluble, and soluble fiber contents of the bread, respectively; TP and DPPH—total polyphenol content and antioxidant activity of bread, respectively; CM—moisture content of the crumb bread; H24 and H72—hardness of the bread crumb 24 and 72 h after baking, respectively; V-100—bread volume per 100 g of bread; OA—overall acceptability of the bread; HI—crumb hardness increase during the storage of the bread; L*—crumb lightness; a* and b*—crumb color parameters; C*—chroma of the crumb; EV—energy value; OMP5, OMP7.5, and OMP10—samples with 5, 7.5, and 10% oyster mushroom powder, respectively; CB—control sample (without OMP).

Principal component analysis (PCA) is a known tool for the analysis of numerical data obtained for a number of objects. In our research, PCA was employed to graphically represent the findings, as shown in Table 2, Table 3, Table 4, Table 5 and Table 6. Figure 2 presents the PCA biplot, constituting a connection of the PCA score plot and a loading plot of two principal components, PC1 and PC2. From the PCA figure, it was possible to indicate which of the tested flour and bread parameters differentiated the tested samples to the greatest extent.

As shown from the PCA graph, PC1 explained the majority (73.91%) of the total variability. It was strongly positively affected by the amylograph peak viscosity (Vmax), the initial temperature of the starch gelatinization (TI), the energy value of the bread (EV), and the carbohydrate (C) and starch (S) contents of the bread, while it was strongly negatively related with all of the parameters from the swelling curve (VA, VB, VC, and Log), the total dietary fiber (TDF) and protein (P) contents of the bread, the water absorption of the flour (WA), and the parameters that describe the antioxidant activity, such as the total polyphenol content (TP) and DPPH activity. PC2, which accounted for 24.69% of the total variability, was strongly positively affected by slowly digestible starch (SDS) and the overall acceptability of the bread (OA), and was negatively related to the bread crumb hardness 24 and 72 h after baking, (H24) and (H72), respectively. On the right side of the plot (positive PC1 values) is positioned the control sample (CB), whereas the OMP10 sample is located on the far-left side (negative PC1 values). The addition of oyster mushroom powder (OMP) enriched the bakery products with protein, dietary fiber, minerals, and polyphenols; at the same time, it caused a decrease in the content of carbohydrates and starch in the finished product. It also increased the activity of enzymes that break down hemicellulose. Therefore, it influenced significant differences in the values of many tested parameters, depending on the percentage share in the 2.5–10% range. For this reason, individual samples are located in different parts of the graph. Only those samples with a share of 5 and 7.5% of OMP addition are located close together. The most useful was the share of OMP, which constituted 7.5%, in terms of the technological parameters and sensory evaluation. Some of the parameters did not increase with the addition of OMP above 7.5%, e.g., in the case of the suspension viscosity at a temperature of 42 °C (VB), or their value decreased, as in the case of the final viscosity (VC). Ultimately, the sensory evaluation was decisive in recognizing the most beneficial OMP content of 7.5%, as it was imperceptible at 5% and unacceptable at 10%.

## 4. Conclusions

The oyster mushroom powder increased the nutritional value of rye flour and its water absorption, also significantly prolonged starch gelatinization and increased the xylolytic activity of the flour. It is important to note that the oyster mushroom powder enriched the bread with dietary fiber, protein, minerals, and phenolic compounds, and increased its antioxidant potential. Notably, adding oyster powder to rye flour had a positive effect on the starch digestion, causing an increase in the share of slowly digestible starch (SDS). It is known that products rich in SDS have a lower glycemic index (GI), so probably the bread with oyster mushrooms will reduce its GI. Adding oyster mushroom powder to rye flour in a proportion not higher than 7.5% did not cause a deterioration in the rye bread’s physicochemical and sensory characteristics. A decrease in the bread volume and crumb hardness, as well as the lower consumer acceptance of the enriched bread, was revealed only when the rye flour was replaced with mushroom powder in the amount of 10%.

## Figures and Tables

**Table 1:** Proximate composition of the rye flour and oyster mushroom powder used for baking.

Parameter/Sample	RF	OMP
M, %	14.98 ± 0.01	10.48 ± 0.18
P, % d.m.	7.03 ± 0.01	22.60 ± 0.06
A, % d.m.	0.72 ± 0.01	6.78 ± 0.04
F, % d.m.	0.95 ± 0.00	1.35 ± 0.01
C, % d.m.	82.40 ± 0.19	25.08 ± 0.47
TDF, % d.m., including:	8.91 ± 0.20	44.19 ± 0.38
S-DF, % d.m.	3.86 ± 0.06	3.51 ± 0.16
IN-DF, % d.m.	5.06 ± 0.13	40.68 ± 0.47
β-Glc, % d.m.	1.48 ± 0.08	–
β-Glf, % d.m.	–	42.08 ± 0.64
α-Gl, % d.m.	–	2.27 ± 0.20
TP, mg GAE/g d.m.	0.68 ± 0.02	4.86 ± 0.05
DPPH, µM Trolox/g d.m.	0.95 ± 0.02	7.92 ± 0.01

RF—rye flour; OMP—oyster mushroom powder; M—moisture content; A—ash content; P—protein content; F—fat content; C—carbohydrate content; TDF—total fiber content; S-DF—soluble fiber content; IN-DF—insoluble fiber content; β-Glc—cereal β-glucan content; β-Glf—fungal β-glucan content; α-Gl—α-glucan content; TP—total polyphenol content; DPPH—antioxidant capacity by the DPPH assay.

**Table 2:** Baking quality of the rye flour and its mixtures with oyster mushroom powder.

Parameter/Sample	RF	RF5	RF7.5	RF10
FN, s	236 ± 1 ^a^	234 ± 3 ^a^	223 ± 3 ^b^	212 ± 1 ^c^
WA, %	61.3 ± 0.1 ^c^	64.0 ± 0.1 ^b^	64.7 ± 0.1 ^a^	65.1 ± 0.1 ^a^
Swelling curve parameters				
VA, AU	105 ± 0 ^c^	220 ± 4 ^b^	225 ± 4 ^b^	270 ± 7 ^a^
VB, AU	105 ± 4 ^c^	200 ± 4 ^b^	270 ± 14 ^a^	270 ± 4 ^a^
VC, AU	100 ± 4 ^d^	180 ± 4 ^c^	235 ± 4 ^a^	200 ± 0 ^b^
Log, -	22 ± 1 ^d^	46 ± 1 ^c^	60 ± 16 ^b^	130 ± 6 ^a^
Amylograph parameters				
T_I_, °C	55.0 ± 0.4 ^a^	54.5 ± 0.4 ^ab^	54.5 ± 0.0 ^ab^	53.5 ± 0.4 ^b^
T_F_, °C	72.0 ± 0.4 ^c^	86.0 ± 0.4 ^b^	88.0 ± 0.4 ^a^	89.5 ± 0.4 ^a^
V_max_, AU	520 ± 4 ^a^	480 ± 7 ^b^	475 ± 4 ^b^	450 ± 4 ^a^

RF—rye flour; RF5, RF7.5, and RF10—rye flour with 5, 7.5, and 10% of oyster mushroom powder; FN—falling number; WA—water absorption; VB—viscosity when the slurry reaches 42 °C; VA—initial viscosity at 30 °C; VC—final viscosity after holding a slurry for 30 min at 42 °C; Log—a logarithmic decrease in the viscosity at 42 °C; T_I_ and T_F_—initial and final temperature of the starch gelatinization, respectively; V_max_—amylograph maximum peak viscosity. The dates marked by the letters ^a–d^ are significantly different (*p* < 0.05); n = 3.

**Table 3:** Physicochemical properties of the control and enriched bread samples.

**Parameter/Sample**		**CB**	**OMB5**	**OMB7.5**	**OMB10**
		Technological value			
V_100_, cm^3^/100 g		195 ± 2 ^a^	195 ± 1 ^a^	193 ± 5 ^a^	185 ± 1 ^b^
M_B_, %		46.7 ± 0.1 ^c^	47.7 ± 0.1 ^b^	48.3 ± 0.3 ^ab^	48.8 ± 0.1 ^a^
H_24_, N		39.4 ± 0.5 ^b^	39.1 ± 0.5 ^b^	34.0 ± 0.5 ^c^	58.6 ± 1.7 ^a^
H_72_, N		68.7 ± 2.1 ^b^	56.9 ± 4.2 ^c^	53.0 ± 2.1 ^c^	78.5 ± 3.1 ^a^
IH, N		29.3 ± 1.6 ^a^	17.8 ± 3.6 ^b^	19.0 ± 2.6 ^b^	19.9 ± 1.4 ^b^
		Color parameters			
Crumb	L*	56.3 ± 1.9 ^a^	49.8 ± 1.8 ^b^	48.9 ± 1.8 ^bc^	48.3 ± 2.0 ^c^
	a*	3.66 ± 1.71 ^c^	5.30 ± 1.80 ^b^	5.50 ± 2.01 ^b^	6.01 ± 1.82 ^a^
	b*	17.00 ± 2.11 ^b^	18.48 ± 1.96 ^b^	20.12 ± 1.91 ^a^	20.12 ± 2.06 ^a^
	C*	17.39 ± 2.02 ^c^	19.22 ± 1.98 ^b^	20.86 ± 2.05 ^ab^	21.00 ± 1.98 ^a^
	ΔE	-	6.89 ± 1.92 ^b^	8.28 ± 2.02 ^ab^	8.87 ± 2.05 ^a^

CB—control bread; OMB5, OMB7.5, and OMB10—bread enriched with 5, 7.5, and 10% of oyster mushroom powder; V_100_—bread volume per 100 g of bread; M_B_—moisture of the bread crumb; H_24_ and H_72_—hardness of the bread crumb 24 and 72 h after baking, respectively; IH—increase in the bread crumb hardness. The dates marked by the letters ^a–c^ are significantly different (*p* < 0.05); n = 3.

**Table 4:** Nutrition composition and antioxidant potential of the control and enriched rye bread.

**Parameter/Sample**	**CB**	**OMB5**	**OMB7.5**	**OMB10**
	Nutrition value			
A_B,_ % d.m.	1.12 ± 0.02 ^c^	1.25 ± 0.01 ^b^	1.42 ± 0.01 ^a^	1.48 ± 0.02 ^a^
P_B_, % d.m.	7.53 ± 0.02 ^d^	8.43 ± 0.01 ^c^	8.94 ± 0.01 ^b^	9.31 ± 0.01 ^a^
F_B_, % d.m.	0.05 ± 0.00 ^c^	0.08 ± 0.01 ^b^	0.12 ± 0.00 ^a^	0.13 ± 0.02 ^a^
C_B_, % d.m.	81.88 ± 0.16 ^a^	79.67 ± 0.05 ^b^	78.53 ± 0.28 ^c^	76.26 ± 0.10 ^d^
S_B_, % d.m_._	72.75 ± 0.03 ^a^	70.24 ± 0.27 ^b^	68.40 ± 0.30 ^c^	66.49 ± 0.25 ^d^
TDF_B_, %	5.03 ± 0.08 ^c^	5.53 ± 0.03 ^b^	5.68 ± 0.15 ^b^	6.57 ± 0.04 ^a^
TDF_B_, % d.m.,	9.43 ± 0.16 ^d^	10.37 ± 0.06 ^c^	11.20 ± 0.28 ^b^	12.82 ± 0.02 ^a^
including: S-DF_B_, % d.m.	3.66 ± 0.16 ^b^	4.26 ± 0.16 ^a^	4.47 ± 0.07 ^a^	4.47 ± 0.05 ^a^
IN-DF_B_, % d.m.	5.77 ± 0.00 ^c^	6.11 ± 0.05 ^c^	6.73 ± 0.28 ^b^	8.35 ± 0.10 ^a^
EV, kcal/100 g	215 ± 1 ^a^	212 ± 1 ^b^	210 ± 0 ^c^	207 ± 1 ^d^
	Antioxidant potential			
TP_B_, mg GAE/100 g d.m.	0.74 ± 0.01 ^d^	1.14 ± 0.05 ^c^	1.35 ± 0.02 ^b^	1.64 ± 0.02 ^a^
DPPH_B_, µM Trolox/g d.m.	1.04 ± 0.02 ^d^	1.51 ± 0.02 ^c^	1.68 ± 0.02 ^b^	1.89 ± 0.01 ^a^

CB—control rye bread; OMB5, OMB7.5, and OMB10—bread enriched with 5, 7.5, and 10% of oyster mushroom powder; A_B_—ash content in the bread; P_B_—protein content in the bread; F_B_—fat content in the bread; C_B_—total carbohydrate content; TDF_B_—total fiber content in the bread; S-DF_B_—content of soluble dietary fiber in the bread; IN-DF_B_—content of insoluble dietary fiber in the bread; TP_B_—content of total polyphenol in the bread; DPPH_B_—bread antioxidant capacity by the DPPH method; EV—energy value of the bread. The dates marked by the letters ^a–d^ are significantly different (*p* < 0.05); n = 3.

**Table 5:** Starch fractions determined by in vitro starch digestion (% starch).

Parameter/Sample	CB	OMB5	OMBB7.5	OMB10
RDS	83.07 ± 0.30 ^a^	79.23 ± 0.77 ^b^	78.97 ± 0.08 ^b^	79.03 ± 0.56 ^b^
SDS	4.19 ± 0.06 ^c^	6.95 ± 0.08 ^b^	8.14 ± 0.22 ^a^	4.03 ± 0.06 ^c^
RS	12.74 ± 0.24 ^b^	13.82 ± 0.69 ^b^	12.89 ± 0.29 ^b^	16.94 ± 0.61 ^a^
SDS + RS	16.93 ± 0.30 ^b^	20.77 ± 0.77 ^a^	21.03 ± 0.08 ^a^	20.97 ± 0.56 ^a^

CB—control bread, OM5, OM7.5, and OM10—bread enriched with 5, 7.5, and 10% of oyster mushroom powder; RDS—rapidly digestible starch; SDS—slowly digestible starch; RS—resistant starch. The dates marked by the letters ^a–c^ are significantly different (*p* < 0.05); n = 3.

**Table 6:** Results of the sensory evaluation of the control and enriched bread samples.

Sample	Color	Aroma	Taste	Texture	Overall Acceptability
CB	7.5 ± 0.4 ^a^	7.6 ± 0.3 ^a^	7.5 ± 0.4 ^b^	8.3 ± 0.1 ^a^	7.5 ± 0.3 ^b^
OMB5	7.7 ± 0.3 ^a^	8.2 ± 0.4 ^a^	8.1 ± 0.3 ^ab^	8.3 ± 0.3 ^a^	8.0 ± 0.3 ^a^
OMB7.5	7.6 ± 0.3 ^a^	8.5 ± 0.4 ^a^	8.6 ± 0.3 ^a^	8.0 ± 0.4 ^a^	8.1 ± 0.2 ^a^
OMB10	5.8 ± 0.4 ^b^	4.5 ± 0.5 ^b^	2.5 ± 0.2 ^c^	5.3 ± 0.1 ^b^	3.5 ± 0.3 ^c^

CB—control bread; OM5, OM7.5, and OM10—bread enriched with 5, 7.5, and 10% of oyster mushroom powder. The dates marked by the letters ^a–c^ are significantly different (*p* < 0.05); n = 3.

## Data Availability

The original contributions presented in the study are included in the article, further inquiries can be directed to the corresponding authors.
